# Metabolic modulation of yogurt fermentation kinetics and acidification by *Bifidobacterium*-starter culture interactions

**DOI:** 10.3389/fmicb.2026.1724590

**Published:** 2026-01-23

**Authors:** Zhi Zhao, Shaoqi Shi, Lele Zhang, Meilun An, Pengcheng Wen, Yue Sang, Haihong Feng, Baochao Hou, Jian He, Wei-Lian Hung, Baolei Li, Liang Zhao, Xiaoxia Li, Ran Wang

**Affiliations:** 1 Key Laboratory of Functional Dairy, Co-constructed by Ministry of Education and Beijing Government, Department of Nutrition and Health, China Agricultural University, Beijing, China; 2 College of Food Science and Engineering, Gansu Agricultural University, Lanzhou, China; 3 Research Center for Probiotics, China Agricultural University, Beijing, China; 4 National Center of Technology Innovation for Dairy, Hohhot, China

**Keywords:** *Bifidobacterium*, fermentation kinetics, metabolomics, post-acidification, probiotic yogurt

## Abstract

**Introduction:**

Probiotic-fortified yogurt has gained substantial consumer preference owing to its well-documented health benefits. However, stability of probiotic yogurt necessitates a comprehensive understanding of microbial dynamics throughout fermentation and storage.

**Methods:**

This study employed an integrated approach combining fermentation kinetics, post-acidification profiling, and untargeted metabolomics to explore the complex interactions between three *Bifidobacterium* strains (*B. animalis* 23426, *B. bifidum* 91, and *B. longum* BB68S) and starter cultures (HYY) during symbiotic fermentation.

**Results:**

The results demonstrate that *Bifidobacterium* supplementation notably enhanced the biomass of *S. thermophilus* (8.13–8.54 lg CFU/mL) after 2 h by upregulating galactose catabolism and riboflavin biosynthesis, thereby reducing fermentation time by 0.5 to 2 h. In contrast, competitive exclusion effects caused a decrease in *L. bulgaricus* biomass by 0.2 to 0.8 log CFU/mL. Over 21-day of refrigerated storage, the acid accumulation in *Bifidobacterium*-enriched yogurts was significantly lower (Δ 3.08–7.49 °T) than in HYY yogurt (Δ 9.42 °T), primarily by downregulation key metabolic pathways involving glycerophospholipid metabolism, branched-chain and aromatic amino acid metabolism, and cofactor biosynthesis, leading to reduced post-acidification.

**Discussion:**

Therefore, *Bifidobacterium* accelerates fermentation by promoting *S. thermophilus* biomass while mitigating post-acidification by inhibiting *L. bulgaricus*. The results provide a scientific basis for developing next-generation probiotic yogurts with controlled acidification profiles and improved shelf-life characteristics.

## 1 Introduction

Fermented dairy products represent a rapidly expanding segment of the global functional food market, with an annual growth rate exceeding 10% due to their distinctive organoleptic properties, nutritional value, and scientifically validated probiotic benefits (Deshwal et al., 2021). The technological and functional characteristics of these products are fundamentally determined by their starter cultures, which govern critical quality parameters including texture development, flavor formation, and bioactive compound production (Ongol et al., 2007; Vénica et al., 2018). The fermentation process in traditional yogurt relies on a microbial consortium consisting of *S. thermophilus* and *L. bulgaricus*. These two bacteria engage in a proto-cooperative relationship that efficiently acidifies the milk and generates characteristic flavor compounds, including acetaldehyde and diacetyl (Deshwal et al., 2021; Zhou et al., 2025; Tian et al., 2017). However, these traditional starters lack targeted probiotic functionalities, prompting increasing interest in developing next-generation cultures incorporating clinically validated probiotic strains from *Lactobacillus* and *Bifidobacterium* genera (Settachaimongkon et al., 2014; Wang et al., 2025).

The scientific rationale for probiotic-enriched yogurts stems from their demonstrated health benefits, which extend beyond basic nutrition to include modulation of gut microbiota composition, immunostimulation, and metabolic regulation (Vénica et al., 2018; Zhang et al., 2024). Clinical evidence indicates that the intake of yogurt containing *B. lactis* BB04 and *L. acidophilus* (Lyofast-LA3) significantly improves acute diarrhea in children (reducing the diarrhea rate from 48 to 8%) (Zahir and Naz, 2022). Additionally, *B. lactis* BB04 demonstrates a beneficial effect on intestinal infections induced by *Listeria monocytogenes* through the production of bifidocin A (Du et al., 2025). In addition to functional enhancement, probiotic strains can enhance fermentation efficiency and product flavor through metabolic cross-talk with traditional starters. For instance, *Lactiplantibacillus plantarum* (*L. plantarum*) WCFS1 accelerates lactose hydrolysis through β-galactosidase activity, reducing fermentation time by 20% compared to conventional cultures (Settachaimongkon et al., 2016; Xue et al., 2024). Similarly, the binary probiotics (*Lactococcus lactis* CGMCC1.5956 and *Lacticaseibacillus casei* CGMCC1.5954) improved amino acid utilization by modulating amino acid metabolism, and increased levels of caproic acid, 2-heptanone, and 2-nonanone, which contributed to a creamy flavor in yogurt (Fan et al., 2024).

Nevertheless, the incorporation of probiotic strains introduces technological challenges, particularly regarding post-acidification during refrigerated storage. While traditional yogurts typically show pH reductions of 0.3–0.5 units over 21 days (Peng et al., 2022), whereas probiotic-enriched variants demonstrate strain-dependent acidification behaviors. Certain *Lacticaseibacillus rhamnosus* (*L. rhamnosus*) GG strains can elevate titratable acidity by up to 45% (Settachaimongkon et al., 2015), while *B. lactis* M8 exacerbates acid production through persistent lactose metabolism, generating acetic acid that drives an additional 0.3 pH unit decrease (Pinto et al., 2017; Wang et al., 2021). Conversely, some strains exhibit acidification-mitigating properties—*L. plantarum* WCFS1 modulates proton-translocating ATPase activity under high NaCl/low pH stress conditions (Settachaimongkon et al., 2016), and *B. lactis* BB12 limits post-acidification to merely 0.1 pH unit reduction over 21 days. This paradoxical situation creates an innovation imperative: developing probiotic yogurts that balance health functionality with technological stability requires precise understanding of strain-specific metabolic behaviors during both fermentation and storage phases.

This study systematically evaluated three selected *Bifidobacterium* strains through co-fermentation with conventional yogurt starter cultures (HYY). The HYY starter consists of *S. thermophilus* and *L. bulgaricus*, which rapidly acidify milk, generate characteristic yogurt flavor compounds (e.g., acetaldehyde and diacetyl), and contribute to gel formation and texture development in yogurt manufacturing. By employing a multi-modal analytical approach, we first characterized the fermentation kinetics through real-time monitoring of titratable acidity, while simultaneously tracking post-acidification profiles during 21-day refrigerated storage to elucidate population dynamics. UHPLC-Q Exactive HF-X analysis was utilized to decipher the metabolic mechanisms. The analysis aimed at revealing the key pathways for organic acid changes during fermentation and storage of these strains, and at clarifying how carbohydrate and distinct amino acid metabolic fluxes affect overall acid accumulation. These results provide critical insights for the rational design of probiotic yogurt formulations, establishing a scientific framework for selecting optimal *Bifidobacterium* strains that balance probiotic functionality with technological stability.

## 2 Materials and methods

### 2.1 Strains, growth media, and inoculation cultures

*B. animalis* 23426 (CGMCC No. 23426), *Bifidobacterium bifidum* (*B. bifidum*) 91 (CGMCC No.23592), *Bifidobacterium longum* (*B. longum*) BB68S (CGMCC No. 14168), and HYY starter cultures (*S. thermophilus* and *L. bulgaricus*) were obtained from the Education-Beijing Municipal Key Laboratory of Functional Dairy Products of China Agricultural University. The three *Bifidobacterium* strains were cultured in commercial De Man, Rogosa, and Sharpe (MRS) medium (Beijing Land Bridge Technology Co., China) under anaerobic conditions at 37 °C. Before the experiments, all strains and starter cultures were stored at −80 °C containing 50% glycerol.

### 2.2 Yogurt manufacture

*B. animalis* 23426, *B. bifidum* 91, or *B. longum* BB68S was activated by three successive transfers in MRS broth at 37 °C, and the inoculum concentration was determined by colony enumeration, adjusted to 7.0 lg CFU/g. Activated cultures were then inoculated into sterile whole milk (protein 32 g/L, fat 38 g/L, carbohydrates 48 g/L, sodium 530 mg/L, calcium 1,000 mg/L) in combination with the commercial starter culture HYY (7.0 lg CFU/g). Control samples contained HYY starter cultures only. The samples were then incubated at 42 ± 1 °C until the titratable acidity reached 70 °T. After fermentation, the samples were stored at refrigeration temperature (4 °C) for 21 days (Midea, Anhui, China). Each group was prepared in triplicate, and all procedures were performed on a sterile, ultra-clean table. Here were the details of the entire yogurt preparation process:

(1) Two hundred milliliters of pasteurized whole milk were pipetted into 500 mL Erlenmeyer flasks. The concentrations of the HYY strain and the three *Bifidobacterium* strains were individually adjusted to 9.0 lg CFU/g using sterile physiological saline.

HYY group: The HYY culture (2 mL) was added to each 500 mL Erlenmeyer flask containing 200 mL whole milk. The mixture was placed in a constant-temperature shaking incubator and shaken at 200 rpm for 30 min. Subsequently, it was evenly aliquoted into 50 mL centrifuge tubes (20 mL per tube), resulting in 10 tubes per flask. Repeat this operation for another two 500 mL Erlenmeyer flasks with 200 mL of whole milk each, adding 2 mL of HYY starter culture to each, followed by thorough stirring and aliquoting. A total of 3 Erlenmeyer flasks were designated as one experimental group.

*B. animalis* 23426 + HYY Group: The HYY culture (2 mL) and *B. animalis* 23426 culture (2 mL) were added to each 500 mL Erlenmeyer flask containing 200 mL whole milk. The remaining aliquoting steps were consistent with those in the HYY group.

*B. bifidum* 91 + HYY Group: The HYY culture (2 mL) and *B. bifidum* 91 culture (2 mL) were added to each 500 mL Erlenmeyer flask containing 200 mL whole milk. The remaining aliquoting steps were consistent with those in the HYY group.

*B. longum* BB68S + HYY Group: The HYY culture (2 mL) and *B. longum* BB68S culture (2 mL) were added to each 500 mL Erlenmeyer flask containing 200 mL whole milk. The remaining aliquoting steps were consistent with those in the HYY group.

(2) The aliquoted samples were placed into an incubator and incubated at 42 ± 1 °C. At the time points of 0, 2, 4, 5, 6, and 7 h of incubation, one 50 mL centrifuge tube (containing 20 mL of sample) was retrieved from each of the 3 replicates in every group. Ten gram of sample was weighed from each centrifuge tube for titratable acidity determination. The incubation was terminated when the sample acidity reached 70 °T.
(3) The remaining 4 tubes of yogurt from each replicate were stored in a refrigerator at 4 °C. Titratable acidity was determined at storage time points of 1, 7, 14, and 21 days.
(4) Repeat Steps 1–3 for 2 independent experimental repetitions.

### 2.3 Viable bacteria count measurement

Viable counts of *S. thermophilus*, *L. bulgaricus*, *B. animalis* 23426, *B. bifidum* 91, and *B. longum* BB68S were determined according to the methods described previously (Ashraf and Shah, 2011). *S. thermophilus* viable counts were determined on M17 agar after 48 h of aerobic incubation at 43 °C. *L. bulgaricus* viable counts were determined on MRS agar pH 5.2 after 72 h anaerobic incubation at 43 °C. The viable counts of *Bifidobacterium* strains were determined on MRS agar (containing LiCl) after 48 h in an anaerobic incubator at 37 °C.

### 2.4 Measurement of pH, titratable acidity

The titratable acidity of set-type yogurt was assessed according to the Chinese national standard GB 5009.239-2016 (Li et al., 2025). A pH electrode (model S210, Mettler Toledo, Zurich, Switzerland) connected to an iCinac System (AMS Alliance, Paris, France) enabled simultaneous pH measurement during titration. The detailed steps and calculation methods for determining the titratable acidity of yogurt are as follows:

A 10.000 g aliquot of well-mixed yogurt was accurately weighed into a 150 mL beaker, followed by the addition of 20 mL of CO₂-free distilled water. Two milliliter of 0.5% phenolphthalein indicator were added to the dispersed sample solution. The 0.1000 mol/L standard NaOH solution was filled into a pre-rinsed burette, and the initial volume reading (V₁, accurate to 0.01 mL) was recorded. Titration was performed at a constant rate of 1 drop per second under continuous magnetic stirring (200 rpm). The titration was stopped immediately when the solution turned a stable pale pink color that persisted for 30 s (this is the visual endpoint of the titration). The final volume reading (V₂, accurate to 0.01 mL) was recorded. Blank Titration: A blank control experiment was conducted simultaneously under identical conditions: 30 mL of CO₂-free distilled water was titrated with the standard NaOH solution, and the volume of NaOH consumed (V₀ = V₂ - V₁, mL) was recorded to correct for interference from indicator impurities and residual CO₂ in water. The titratable acidity was calculated according to the following formula:

X=(V−V0)c×100/(m×0.1)

Where:

X: the acidity of the specimen, °T [calculated based on 0.1 mol/L sodium hydroxide consumed by 100 g sample, mL/100 g];

V: Volume of standard NaOH solution consumed by the sample (mL) = V2 − V1;

V0: The volume of the sodium hydroxide standard solution consumed for the blank test, mL;

c: Concentration of standard NaOH solution (mol/L);

100: 100 g specimen;

m: Mass of the yogurt sample (g).

### 2.5 Determination of the non-volatile components using UHPLC-Q Exactive HF-X

Non-volatile metabolites were isolated and characterized following the methodology outlined by Li X. et al. (2024). Metabolite extraction utilized 0.02 mg/mL L-2-chlorophenylalanine as the internal standard. A mixture of 50 mg lyophilized material and 400 μL methanol was homogenized using a high-throughput tissue pulverizer (Wonbio-96c, Shanghai Wanbo Biotechnology Co., Ltd.). Sample disruption involved grinding at 50 Hz for 6 min, followed by sonication at 40 kHz for 30 min, and centrifugation at 12,000 rpm for 15 min at 4 °C. The supernatant was transferred for LC–MS/MS analysis. It is notable that all steps were executed under low-temperature conditions to facilitate protein solidification.

Mass spectral data were acquired using a UHPLC-Q Exactive HF-X system (Thermo Fisher Scientific, United States). The mobile phase A was 0.1% formic acid (v/v) in acetonitrile. The mobile phase B was 47.5% acetonitrile (v/v), and 47.5% acetonitrile isopropanol (v/v) in ultrapure water. The solvent gradient changed according to the following conditions: from 0 to 3.5 min, 0% B to 24.5% B (0.4 mL/min); from 3.5 to 5 min, 24.5% B to 65% B (0.4 mL/min); from 5 to 5.5 min, 65% B to 100% B (0.4 mL/min); from 5.5 to 7.4 min, 100% B to 100% B (0.4 mL/min to 0.6 mL/min); from 7.4 to 7.6 min, 100% B to 51.5% B (0.6 mL/min); from 7.6 to 7.8 min, 51.5% B to 0% B (0.6 mL/min to 0.5 mL/min); from 7.8 to 9 min, 0% B to 0% B (0.5 mL/min to 0.4 mL/min); from 9 to 10 min, 0% B to 0% B (0.4 mL/min) for equilibrating the systems. The sample injection volume was 2 μL and the flow rate was 0.4 mL/min. The column temperature was maintained at 40 °C. During the analysis period, all the samples were stored at 4 °C.

### 2.6 Statistical analysis

Comprehensive details on the screening, normalization, and analysis of the LC–MS data are available in our prior research (Li X. et al., 2024). The pretreatment of LC/MS raw data was performed by Progenesis QI software, generating a three-dimensional data matrix in CSV format. The data matrix derived from the database search was uploaded to the Majorbio cloud platform^1^ for further data analysis. Firstly, the data matrix underwent pre-processing, following these steps: At least 80% of the metabolic features detected in any set of samples were retained. After filtering, for specific samples exhibiting metabolite levels below the lower limit of quantification, the minimum metabolite value was estimated, and each metabolic signature was normalized to the sum. To mitigate errors arising from sample preparation and instrument instability, the response intensities of the sample mass spectrometry peaks were normalized using the sum normalization method, yielding the normalized data matrix. Meanwhile, variables from quality control (QC) samples exhibiting a relative standard deviation (RSD) greater than 30% were excluded and log10-transformed to achieve the final data matrix for subsequent analysis. All sample metabolomic data were screened and preprocessed using MetaboAnalyst.^2^ Data visualization was achieved using principal component analysis (PCA). Identification of small molecules from the LC–MS data was performed using Progenesis QI software, which conducted automatic queries of the Human Metabolome Data Bank (HMDB), ChemSpider, and the METLIN database. The metabolomic data were processed with a mass precision of 10 ppm and a statistical significance threshold (*p*-value) of 0.05. Mummichog analysis was conducted using GraphPad Prism 9 to assess the significance (*p* < 0.05) of data differences, with experimental data were expressed as means ± standard deviation (SD).

## 3 Results and discussion

### 3.1 Fermentation kinetics and acidification profiles

**Figure 1:**
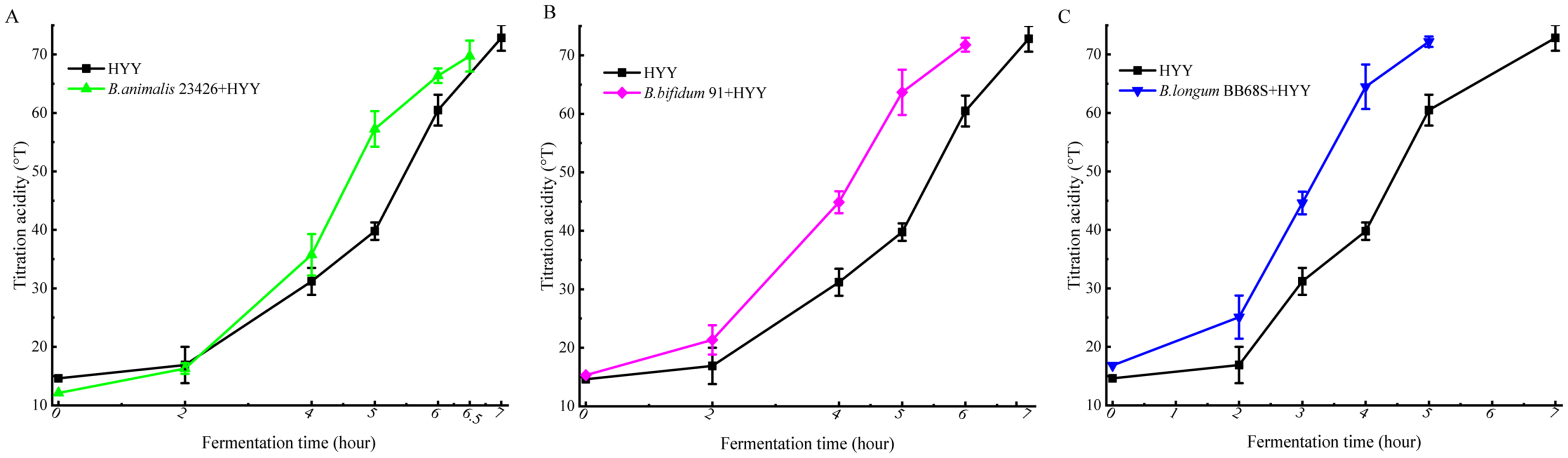
Acidification kinetics of co-fermentation systems containing different *Bifidobacterium* strains with commercial yogurt starter culture (HYY). **(A)** *B. lactis* 23426. **(B)** *B. bifidum 91*. **(C)** *B. longum BB68S*. All *bifidobacterial* strains were inoculated at 1 × 10^7^ CFU/mL in aseptic pure milk with HYY starter culture (50 g/ton). Data points represent mean values of titratable acidity (°T) with error bars indicating standard deviation (*n* = 3 biological replicates).

Figure 1 illustrates the strain-specific acidification profiles of three *Bifidobacterium* strains (1 × 10^7^ CFU/mL). *Bifidobacterium* supplementation accelerated acid production after 2 h, shortening the time to reach the end of fermentation (70 °T). Additionally, the results demonstrated the differential impacts of these strains on fermentation kinetics when comparing control yogurts to those co-fermented with *Bifidobacterium*. Specifically, *B. lactis* 23426 significantly increased the acidification level (from 19.60 ± 3.11 °T to 31.21 ± 2.31 °T) at 2–4 h, and triggered a 0.5 h shorter fermentation endpoint (Figure 1A) (*p* < 0.05). Moreover, the yoghurt samples supplemented with *B. bifidum* 91 and *B. longum* BB68S exhibited a significantly increased 2–4 h acidification level (from 21.35 ± 2.51 °T to 41.89 ± 1.88 °T for *B. bifidum* 91 (Figure 1B) and from 20.09 ± 3.68 °T to 64.48 ± 3.80 °T for *B. longum* BB68S) (Figure 1C) and shortened fermentation endpoint time by 1 h and 2 h, respectively. Acid production capacity is a pivotal determinant in industrial yogurt manufacturing, directly influencing fermentation efficiency and economic viability (Zare et al., 2012). In industrial yogurt production, even a 0.5 h reduction in fermentation time can increase daily production capacity and lower energy consumption, thereby improving processing efficiency and reducing variability between batches. An increase in the production of organic acids—including lactic and acetic acid—is positively correlated with acidity, which not only influences product texture but also serves as a critical indicator for determining the fermentation endpoint (Settachaimongkon et al., 2014; Sun et al., 2024) demonstrated that incorporating *Bifidobacterium* M-8 into yogurt fermentation starters notably elevated the concentrations of acetic, lactic, citric, succinic, and tartaric acids, consequently shortening the fermentation duration. Tian et al. (2022) demonstrated that *B. longum* CCFM5871 accelerates fermentation by employing the Leloir pathway and galactose-associated genes GH2 to boost galactose metabolism, thereby increasing viable bacterial counts at fermentation’s end. Therefore, depending on the strain, the addition of *Bifidobacteria* improves fermentation by boosting the growth and viability of yogurt cultures, though their efficacy in doing so varies.

### 3.2 Microbial community dynamics during active fermentation

**Figure 2:**
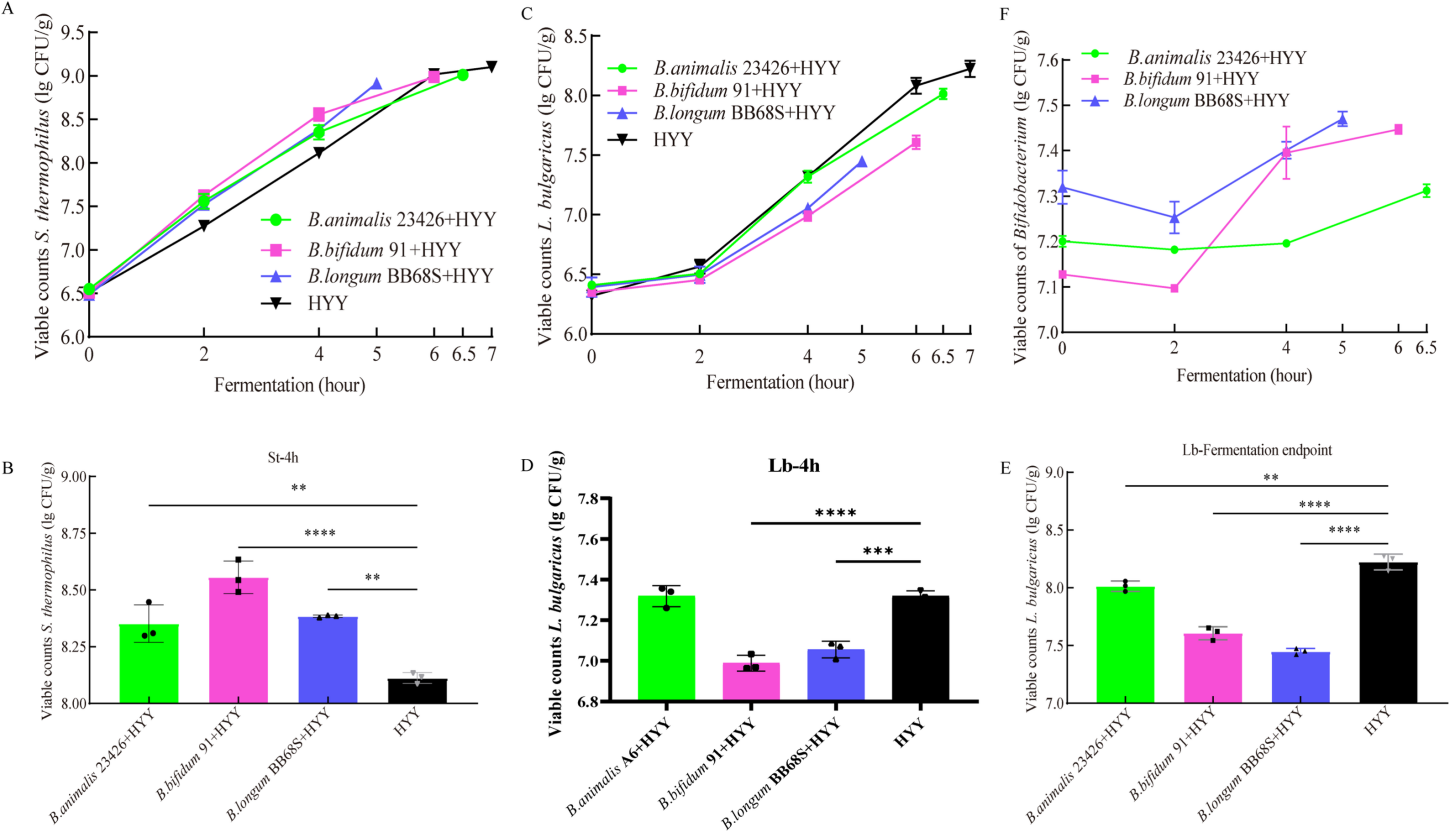
Dynamics of viable bacterial counts during set-yogurt fermentation. **(A)** Growth kinetics of *S. thermophilus* throughout fermentation. **(B)** Comparative analysis of *S. thermophilus* viable counts at the 4 h fermentation time point. **(C)** Growth profile of *L. bulgaricus* during fermentation. **(D)** Comparison of *L. bulgaricus* cell densities at the 4 h fermentation time point. **(E)** Final viable counts of *L. bulgaricus* at fermentation endpoint (70°T). **(F)** Growth curves of *Bifidobacterium* strains (*B. lactis* 23426, *B. bifidum* 91, and *B. longum* BB68S) during co-fermentation. Error bars represent the standard deviation of triplicate measurements (*n* = 3). Colony counts were determined by plate counting on appropriate selective media after serial dilution. **p < 0.01, ***p < 0.001, and ****p < 0.0001.

The growth of *S. thermophilus, L. bulgaricus,* and three species of *Bifidobacterium* was monitored during fermentation, concerning the differential media studied by Ashraf and Shah (2011). The introduction of three *Bifidobacterium* strains maintained stable final populations of *S. thermophilus* (8.9–9.1 log CFU/mL) at fermentation endpoint (Figure 2A). Notably, a transient 1.3-fold proliferation surge in *S. thermophilus* was observed at the 4-h (*p* < 0.05, Figure 2B), suggesting temporary metabolic synergy that ultimately reduced total fermentation time by 22%. In contrast, all *Bifidobacterium* co-cultures significantly suppressed *L. bulgaricus* viability after 2 h of fermentation (Figure 2C). Compared to the HYY group, the yogurt samples fermented with 91 + HYY and BB68S + HYY showed a significant difference in the viability of *L. bulgaricus* at 4 h of fermentation (Figure 2D). Specifically, the 91 + HYY group had 6.9 lg CFU/mL (vs. control 7.3 lg CFU/mL, *p* < 0.0001), while the BB68S + HYY group had 7.0 lg CFU/mL (vs. control 7.3 lg CFU/mL, *p* = 0.0002). At fermentation endpoint, quantification revealed marked reductions in *L. bulgaricus* counts versus HYY control at fermentation endpoint (8.2 lg CFU/mL): 23426 + HYY (8.0 lg CFU/mL, *p* < 0.01), 91 + HYY (6.9 lg CFU/mL, *p* < 0.0001), and BB68S + HYY (7.0 lg CFU/mL, *p* < 0.0001) groups (Figure 2E).

During co-fermentation, the three *Bifidobacterium* strains demonstrated analogous growth dynamics, characterized by an initial viability decline followed by robust proliferation, ultimately viable cell counts were 7.31 lg CFU/mL (23426), 7.44 lg CFU/mL (91), and 7.46 lg CFU/mL (BB68S) (Figure 2F). The transient reduction in *Bifidobacterium* viability during the first 2 h is likely attributable to residual dissolved oxygen in the fermentation matrix (Turgut and Diler, 2023). Subsequent recovery coincided with oxygen depletion mediated by *S. thermophilus* metabolism and lactose hydrolysis-derived galactose availability, which enhanced β-galactosidase activity in *Bifidobacterium* to facilitate anaerobic proliferation (Fischer and Kleinschmidt, 2021). Notably, this proliferative phase synchronized precisely with both the exponential growth of *S. thermophilus* and the onset of *L. bulgaricus* growth inhibition. The *Bifidobacterium*-mediated stimulation of *S. thermophilus* biomass accumulation accelerated acidification kinetics, effectively reducing total fermentation duration. Furthermore, *Bifidobacterium* metabolic byproducts, specifically acetate accumulation, disrupted *L. bulgaricus* membrane integrity through proton motive force destabilization, establishing competitive microbial equilibrium within the consortium (Meng et al., 2021).

### 3.3 Metabolic fingerprinting of *bifidobacteria*-enriched yogurts during fermentation

**Figure 3:**
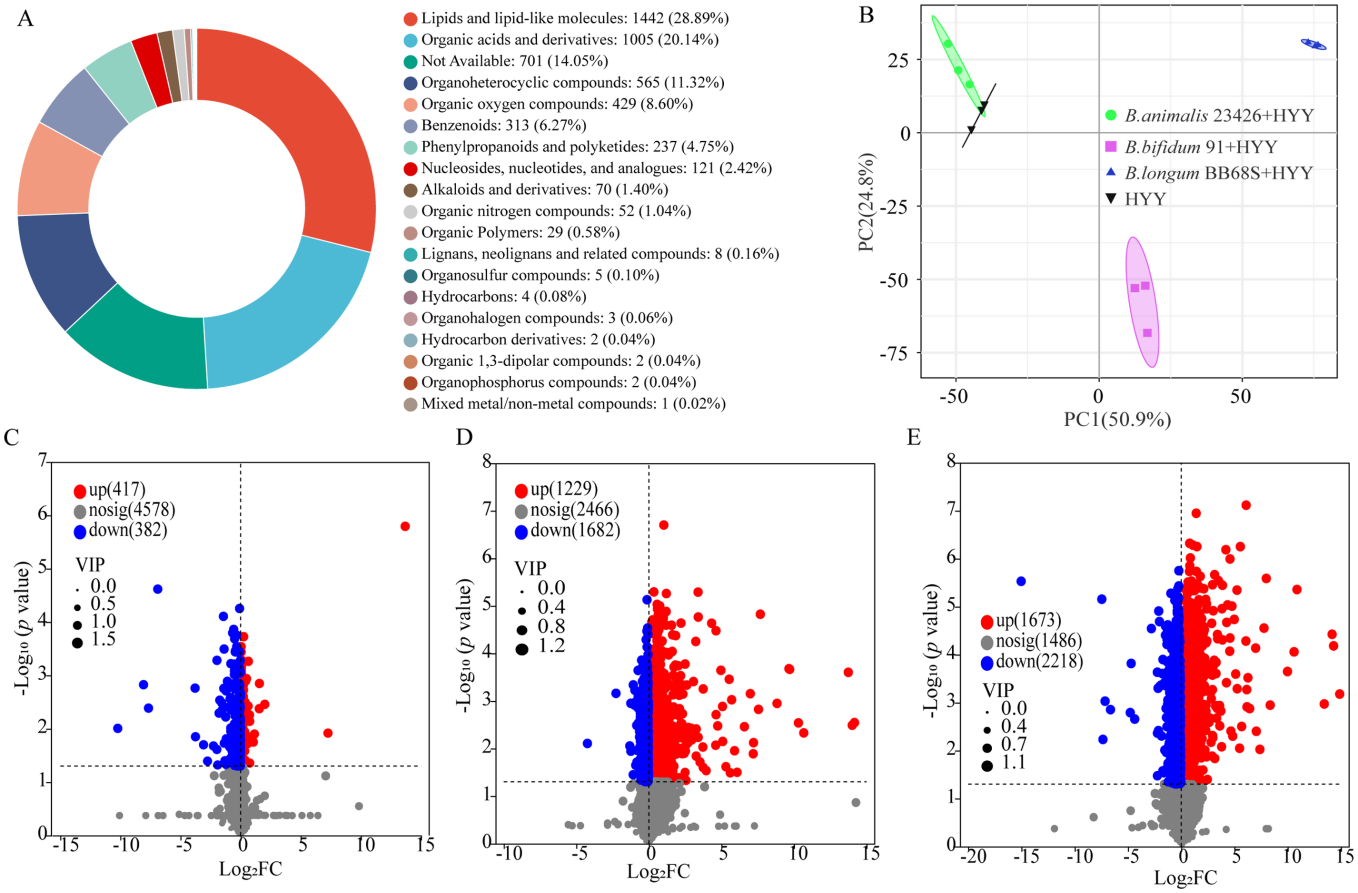
Metabolomic profiling of non-volatile metabolites in *Bifidobacterium*-yogurt starter co-cultures at fermentation endpoint. **(A)** Chemical classification of identified metabolites based on HMDB taxonomy. **(B)** PCA score plot showing metabolic profile clustering among different fermentation groups. **(C–E)** Volcano plots visualizing significantly differential metabolites between: **(C)** 23426 + HYY vs. HYY, **(D)** 91 + HYY vs. HYY, and **(E)** BB68S + HYY vs. HYY. Metabolite identification was performed using UHPLC-Q Exactive HF-X with both positive and negative ionization modes, and data were normalized to internal standards and unit variance-scaled before multivariate analysis. Differential metabolites were identified using the following thresholds: VIP score >1 from OPLS-DA/PLS-DA, statistical significance (*p* < 0.05 by Student’s *t*-test), and absolute log₂ fold-change >1 (equivalent to FC > 2 or FC < 0.5). All analyses were performed using unit variance-scaled data from triplicate biological replicates.

At the end of fermentation, 5,043 metabolites were identified with high confidence based on accurate mass measurement, MS/MS fragmentation matching, and database searches (HMDB, METLIN, ChemSpider). To probe the metabolic alterations induced by different *Bifidobacteria* strains in yogurt, an untargeted metabolomic analysis of three enriched products revealed 5,043 quantified metabolites, categorized as follows: lipids and lipid-like molecules (1,642), organic acids (1,207), organoheterocyclic compounds (716), organic oxygen compounds (629), benzenoids (313), phenylpropanoids and polyketides (237), Nucleosides, nucleotides, and analogs (121), alkaloids and derivatives (70), and other compounds (108) (Figure 3A; Supplementary Table S1). This high-coverage detection substantially exceeds prior reports in fermented dairy research, exemplified by (Wang et al., 2025)'s identification of merely 215 metabolites in selenium-enriched *L. plantarum* NML21 yogurt using UHPLC–MS/MS methodology. The analytical depth achieved through UHPLC-Q Exactive HF-X platforms validates untargeted metabolomics as a powerful discovery tool for mapping complex biochemical transformations—including amino acid flux, vitamin biosynthesis, and lipid remodeling—under probiotic-modulated fermentation regimes (Fan et al., 2023; Peng et al., 2022). This expansive metabolite inventory establishes a critical foundation for mechanistically elucidating *Bifidobacterium*-driven metabolic adaptations in yogurt matrices.

PCA was applied to non-volatile metabolite profiles derived from three *bifidobacteria*-supplemented yogurt treatments—*B. animalis* 23426 (23426 + HYY), *B. bifidum 91* (91 + HYY), and *B. longum* BB68S (BB68S + HYY)—alongside the starter cultures (HYY). The PCA score plot demonstrated tight clustering of triplicate biological replicates (Figure 3B), confirming methodological reproducibility and data reliability (Li Y. et al., 2024). The combined variance explained by PC1 and PC2 amounted to 75.7% of the total (50.9 and 24.8%, respectively), as illustrated in Figure 3B. Notably, the 23426 + HYY treatment clustered adjacent to the HYY control along both principal components, while 91 + HYY and BB68S + HYY samples formed distinct, well-separated clusters. This differential distribution signifies conserved metabolite signatures between *B. animalis* 23426-enriched and control yogurts, whereas *B. bifidum* 91 and *B. longum* BB68S induced significant metabolomic divergence. These strain-specific metabolic profiles align with prior observations by (Fan et al., 2022), wherein supplementation with *Levilactobacillus brevis* CGMCC1.5954 and *Lacticaseibacillus casei* CGMCC1.5956 generated similarly discrete PCA separation patterns. These findings substantiate that individual probiotic strains exert unique and quantifiable influences on yogurt metabolic landscapes.

To delineate strain-specific impacts of *Bifidobacterium* supplementation on fermented milk metabolomes, we conducted differential abundance analysis through volcano plot visualization. Metabolites were deemed significantly altered based on variable importance in projection (VIP ≥ 1) and *p* < 0.05. Comparative analysis revealed substantial metabolic reprogramming: the 23426 + HYY yogurt exhibited 799 differentially abundant metabolites versus HYY (417 upregulated, 382 downregulated; Figure 3C). More pronounced alterations occurred in 91 + HYY yogurt with 2,911 differential metabolites (1,229 upregulated, 1,682 downregulated; Figure 3D), while BB68S + HYY yogurt induced the most extensive remodeling (3,891 differential metabolites; 1,673 upregulated, 2,218 downregulated; Figure 3E). Across all comparisons, dominant differential metabolite subclasses included amino acids, peptides and analogs, carbohydrates, and fatty acids. While (Wang et al., 2025) documented selenium-enriched *L. plantarum* NML21’s modulation of amino acid and carbohydrate metabolism, our findings demonstrate that *Bifidobacterium* strains additionally exert significant influence on fatty acid metabolic pathways, indicating broader regulatory capacity in fermented dairy systems.

### 3.4 Pathway analysis of differentially regulated metabolites during fermentation

**Figure 4:**
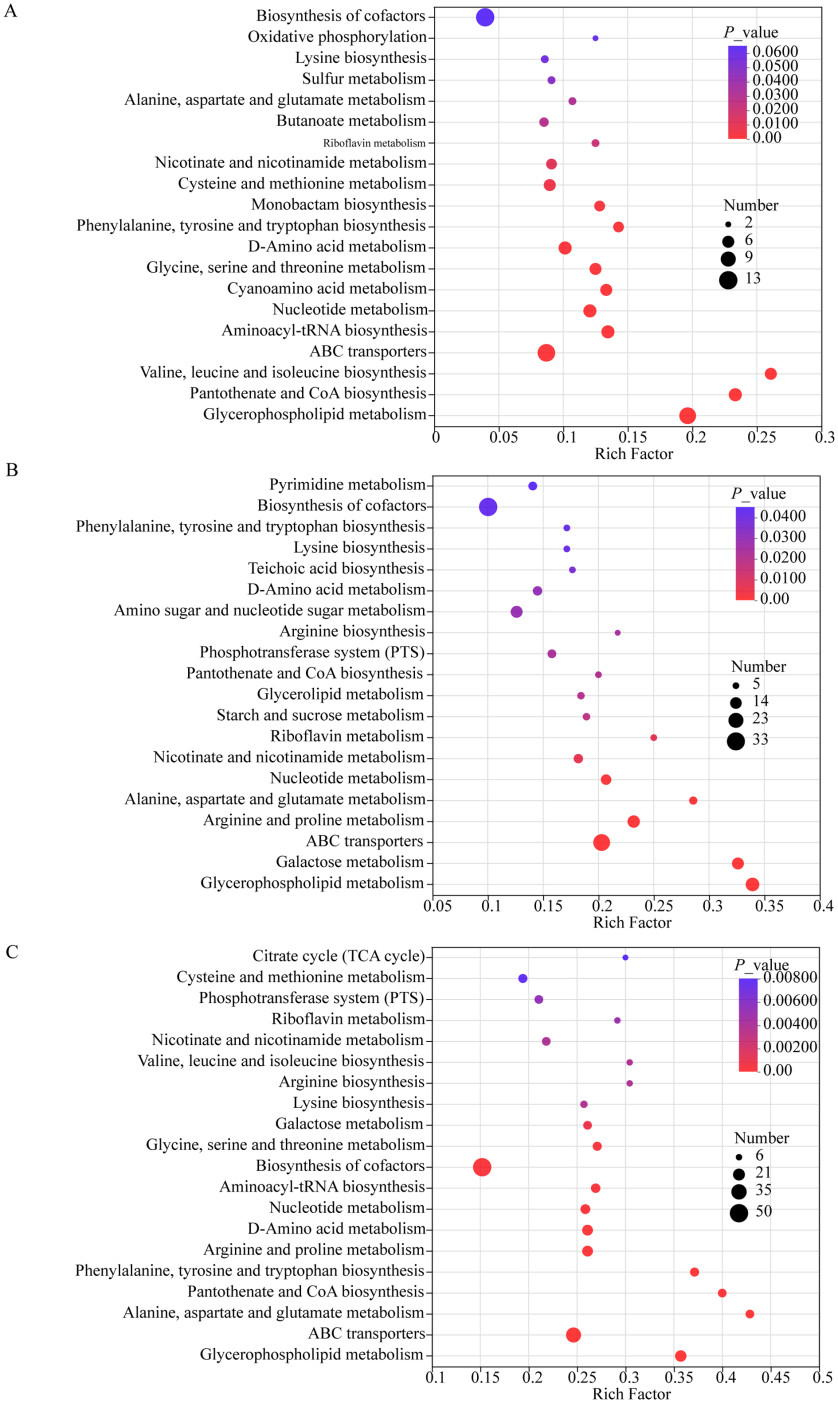
Metabolic pathway enrichment analysis of differentially expressed metabolites at fermentation endpoint. **(A–C)** Pathway impact analysis comparing *Bifidobacterium*-enriched yogurts to HYY yogurt, highlighting significantly enriched metabolic pathways (*p* < 0.05) in both positive and negative ionization modes: **(A)** 23426 + HYY vs. HYY, **(B)** 91 + HYY vs. HYY, and **(C)** BB68S + HYY vs. HYY. All analyses were performed using MetaboAnalyst 5.0 with the KEGG pathway database, considering metabolites with VIP > 1.0 and *p* < 0.05 as statistically significant. The x-axis represents the pathway impact score calculated based on topological centrality, while the y-axis shows −log_10_ (*p*-value) from Fisher’s exact test. The bubble size represents pathway impact value, while color intensity indicates −log_10_ (*p*-value).

To delineate strain-specific metabolic features and pathways induced by *Bifidobacterium* supplementation relative to starter cultures at fermentation termination, pathway topology analysis was performed employing MetaboAnalyst’s hypergeometric distribution algorithm. A more comprehensive characterization of the relative importance of pathways in the overall network was achieved by employing Relative Betweenness Centrality analysis. Distinct perturbations in metabolic pathways were revealed through comparative metabolomics. Significant alterations were identified in the 23426 + HYY vs. HYY comparison (Figure 4A), including metabolism of pyruvate; glycine, serine, and threonine; valine, leucine, and isoleucine biosynthesis; C5-branched dibasic acid; and riboflavin. For the 91 + HYY vs. HYY comparison, six pathways featuring significant non-volatile components were found (Figure 4B), such as teichoic acid biosynthesis, starch and sucrose metabolism, alanine, aspartate, and glutamate metabolism, glycerophospholipid metabolism, galactose metabolism, and riboflavin metabolism. In the analysis of BB68S + HYY versus HYY, seven pathways showed differential regulation (Figure 4C), encompassing teichoic acid biosynthesis, alanine, aspartate, and glutamate metabolism, phenylalanine, tyrosine, and tryptophan biosynthesis, glycerophospholipid metabolism, nucleotide metabolism, galactose metabolism, and riboflavin metabolism. This comparative evidence substantiates that probiotic strain specificity fundamentally governs metabolic pathway redirection during fermentation, thereby modulating both temporal dynamics and functional outcomes in yogurt production. Modifications to phenylalanine metabolism, glycine, serine, threonine metabolism, and the glycerophospholipid pathway have been primarily associated with the activity of *L. plantarum* CCFM8610, as evidenced by previous studies (Huang et al., 2022). While *L. plantarum* P9 predominantly altered fatty acid biosynthesis, degradation, phenylalanine metabolism, proteolysis, and amino acid metabolism (Guo et al., 2024).

The metabolic pathways modulated by *B. animalis* 23426 supplementation demonstrate distinct functional specialization toward microbial survival and proliferation mechanisms. For example, *Bifidobacterium* species utilize the threonine pathway for the biosynthesis of vitamin B12 precursors, while energy production is facilitated by pyruvate metabolism (Rätsep et al., 2024). In particular, flavin mononucleotide and flavin adenine dinucleotide coenzymes, which are derived from riboflavin (vitamin B₂) metabolism, serve as critical components of the energy metabolism machinery of *Bifidobacterium* (Bermúdez-Humarán et al., 2024). In stark contrast, supplementation with either *B. bifidum* 91 or *B. longum* BB68S predominantly activates galactose catabolic pathways—a metabolic signature distinct from conventional starter cultures (*S. thermophilus* and *L. bulgaricus*) which preferentially metabolize the glucose moiety during lactose hydrolysis, leading to characteristic galactose accumulation (Yang et al., 2023). *Bifidobacterium*-mediated galactose utilization occurs through two coordinated biochemical routes: (1) the Leloir pathway (catalyzed by the *galKTE* genes) for galactose assimilation and (2) the phosphoketolase pathway for subsequent fermentation (Tian et al., 2022). This dual-pathway strategy enables efficient conversion of residual galactose into lactate and acetate, thereby accelerating acidification kinetics and significantly reducing fermentation duration across all three *Bifidobacterium*-supplemented systems (23426, 91, and BB68S).

### 3.5 Acidification patterns and post-fermentation microbial stability during refrigerated storage

**Figure 5:**
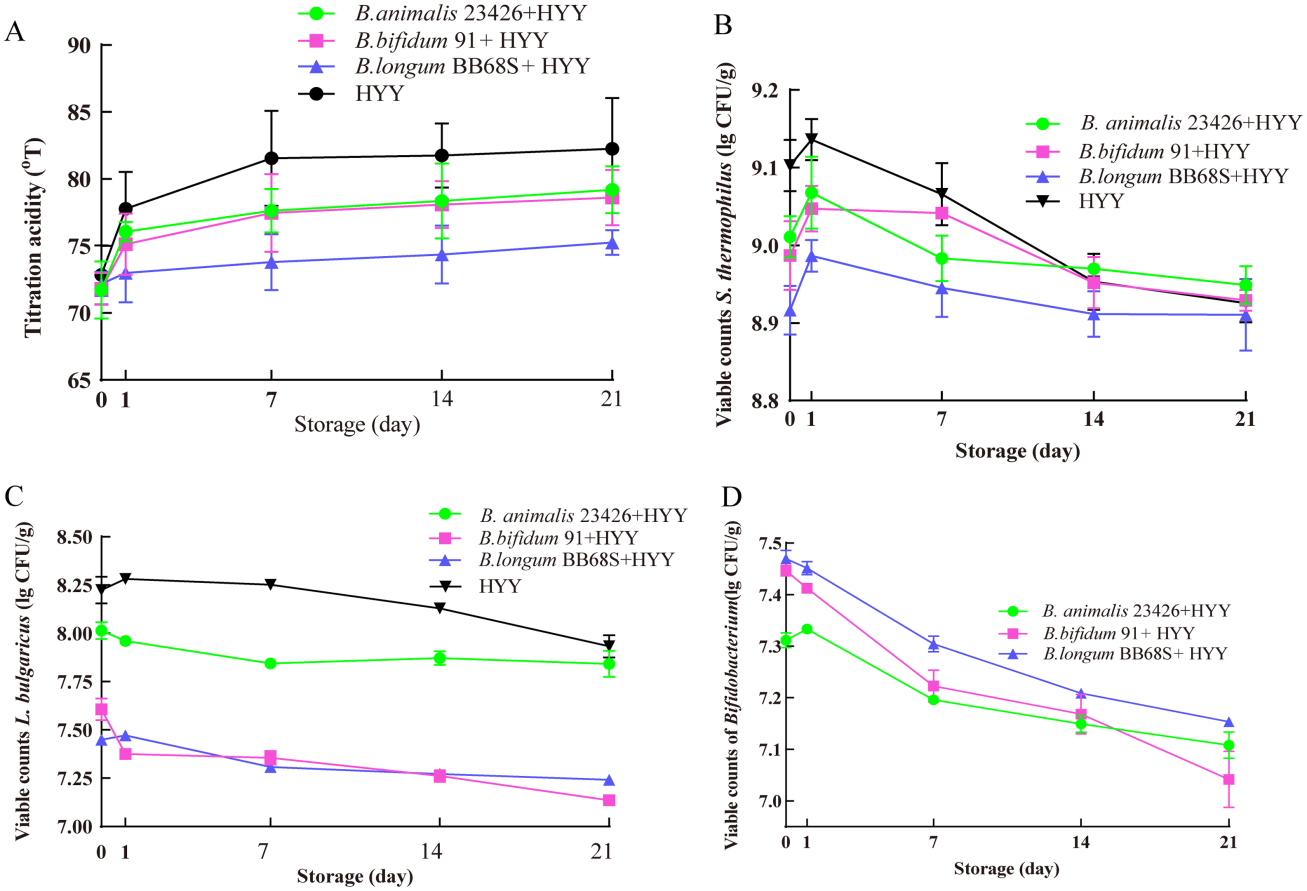
Microbial viability and acidification kinetics during refrigerated storage (4 °C) of yogurt co-fermented with *Bifidobacterium* strains. **(A)** Acidification kinetics of *Bifidobacterium* strains with starter cultures during storage. **(B–D)** Viable cell counts (log_10_ CFU/mL) of **(B)** *S. thermophilus*, **(C)** *L. bulgaricus*, and **(D)** *Bifidobacterium* strains during 21-day refrigerated storage. Data points represent mean values from triplicate experiments with error bars indicating standard deviation (*n* = 3). Colony counts were determined by plate counting on appropriate selective media after serial dilution.

Figure 5 depicts the evolution of acidity parameters, probiotic viability, and starter cultures’ stability in yogurt samples during 21-day refrigerated storage (4 °C). All formulations exhibited progressive titratable acidity (TA) elevation, with maximal acidification occurring during days 0–7 (Figure 5A). Post-storage analysis revealed that BB68S + HYY yogurt demonstrated significantly attenuated TA accumulation (3.08 ± 0.57 °T) versus HYY yogurt (9.42 ± 1.25 °T; *p* < 0.05). Although *B. animalis* 23426 (7.49 ± 0.49 °T) and *B. bifidum 91* (6.82 ± 1.87 °T) supplements showed non-significant TA reduction relative to HYY yoghurt (*p* > 0.05), both exhibited moderating trends, indicating *bifidobacterial* supplementation suppression of post-acidification in yogurt.

*S. thermophilus* viability remained stable (>8.9 log CFU/g) across all groups, regardless of *bifidobacterial* supplementation, with no significant intergroup differences observed (*p* > 0.05) (Figure 5B). In contrast, *L. bulgaricus* viability displayed significant intergroup divergence, reflecting initial fermentation endpoint differences. BB68S + HYY and 91 + HYY formulations consistently maintained lower *L. bulgaricus* counts versus HYY (*p* < 0.05), while 23426 + HYY showed significant reduction only during days 0–7 (Figure 5C). All *bifidobacterium* strains underwent progressive viability decline (Figure 5D), likely due to cumulative oxidative-acidic stress. These observations corroborate Sarn et al.’s findings wherein *L. plantarum* WCFS1 and *B. animalis* BB-12 pretreatment impaired *L. bulgaricus* survival, mitigating post-acidification, while *L. rhamnosus* GG enhanced acidification via viability preservation (Settachaimongkon et al., 2016; Settachaimongkon et al., 2015). Although *L. bulgaricus*, with its inherent acid tolerance, maintained metabolic activity through efficient utilization of available carbon sources, facilitating sustained post-acidification (Yue et al., 2022), *bifidobacteria* modulate post-acidification intensity through strain-specific inhibition of *L. bulgaricus*, potentially mediated by competitive exclusion or antimicrobial metabolite production. These findings provide practical insights for the application of *bifidobacteria* in dairy products, highlighting the importance of *bifidobacteria* as auxiliary strains—these strains not only enhance fermentation performance but also improve product stability during refrigerated storage. Dairy manufacturers can extend shelf life, improve sensory quality, and introduce functional yogurt products that meet consumer expectations by optimizing starter culture formulations and monitoring key metabolic markers.

### 3.6 Metabolic fingerprinting of *Bifidobacterium*-enriched yogurts during storage

**Figure 6:**
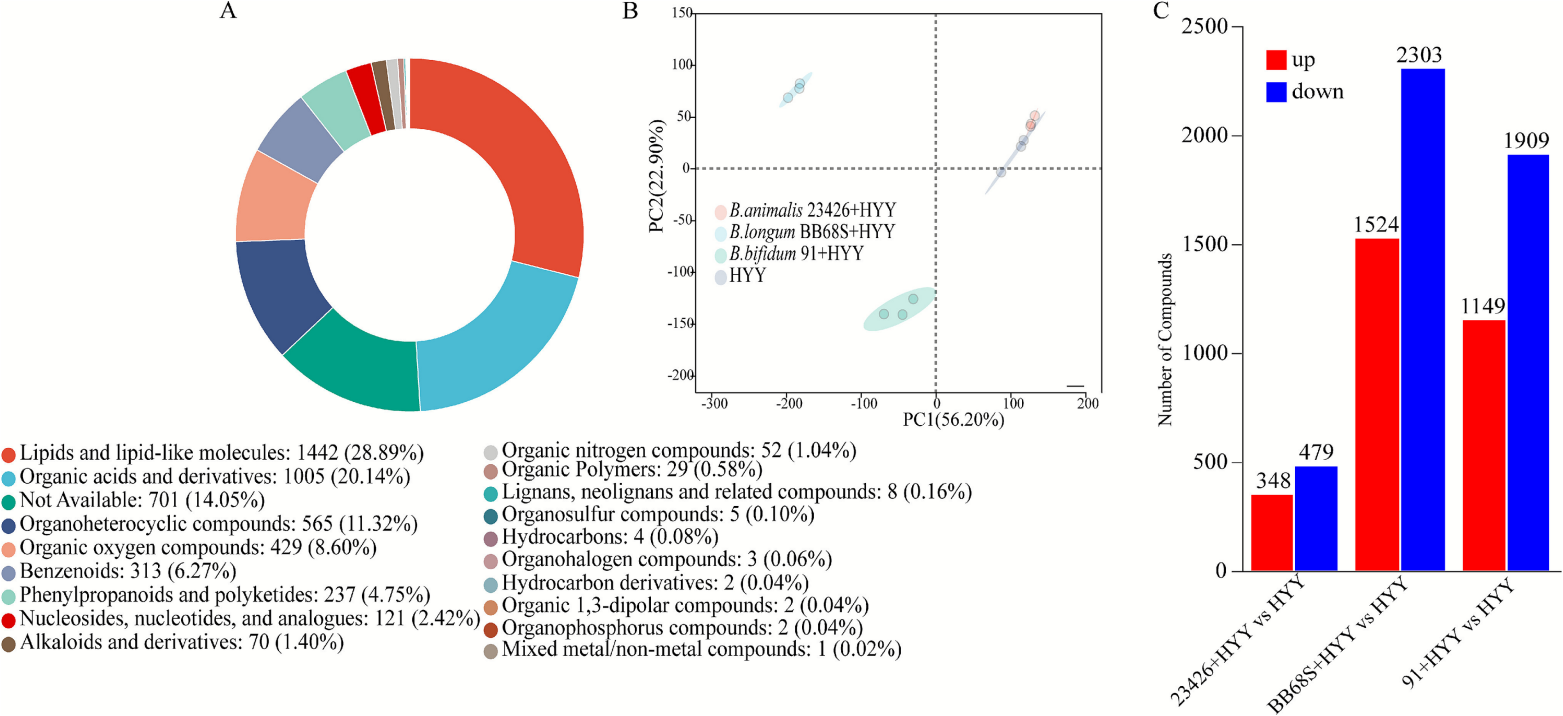
Metabolomic profiling of *Bifidobacterium* co-fermented yogurts during refrigerated storage. **(A)** Chemical classification of metabolites based on HMDB taxonomy after 7-day storage at 4 °C. **(B)** PCA score plot demonstrating metabolic profile segregation among treatment groups. **(C)** Bar chart visualization of significantly altered metabolites (VIP > 1.0, *p* < 0.05 by Student’s *t*-test, and FC > 2 or FC < 0.5) in probiotic-enriched yogurts compared to conventional yogurt after 7-day cold storage. Metabolite identification was performed using UHPLC-Q Exactive HF-X with both positive and negative ionization modes, and data were normalized to internal standards and unit variance-scaled before multivariate analysis. Differential metabolites were identified using the following thresholds: VIP score >1 from OPLS-DA/PLS-DA, statistical significance (*p* < 0.05 by Student’s *t*-test), and absolute log₂ fold-change >1 (equivalent to FC > 2 or FC < 0.5). All analyses were performed using unit variance-scaled data from triplicate biological replicates.

Metabolomic profiling of yogurt samples following a 7-day refrigeration period at 4 °C resulted in the identification of 5,011 metabolites. The composition of this metabolome included: 1,442 lipids and lipid-like molecules, 1,005 organic acids, 565 organoheterocyclic compounds, 429 organic oxygen compounds, 313 benzenoids, 237 phenylpropanoids and polyketides, 121 nucleosides, nucleotides, and analogs, 70 alkaloids and derivatives, and 809 compounds that remained unclassified (Figure 6A; Supplementary Table S2). PCA of non-volatile metabolites demonstrated storage-stable clustering patterns that mirrored fermentation endpoint distributions (Figure 6B), indicating that *Bifidobacterium* strain selection fundamentally determines yogurt metabolite composition. Different adjunct strains are likely to induce distinct shifts in metabolic pathways, and such profiling can guide the selection of strains with desired functional traits, such as targeted flavor enhancement, improved texture, or increased nutritional value.

To systematically investigate the association between *Bifidobacterium*-induced metabolic alterations and post-acidification phenomena in yogurt, we performed comprehensive differential metabolite analysis using volcano plot visualization. Selection of metabolites was performed using a VIP score cutoff of 1 and a statistical significance threshold of *p* < 0.05. Comparative analysis revealed substantial strain-dependent metabolic variations: (1) 23426 + HYY yoghurt showed 872 differentially expressed metabolites (348 up-regulated, 479 down-regulated; Figure 6C); (2) BB68S + HYY yoghurt exhibited 3,827 differential metabolites (1,524 up-regulated, 2,303 down-regulated; Figure 6C); and (3) 91 + HYY yoghurt demonstrated 3,058 metabolic alterations (1,149 up-regulated, 1,909 down-regulated; Figure 6C). Notably, the 23426 + HYY group displayed significantly fewer metabolic perturbations compared to other *Bifidobacterium*-supplemented groups.

### 3.7 Metabolic pathway remodeling during cold storage of probiotic yogurts

**Figure 7:**
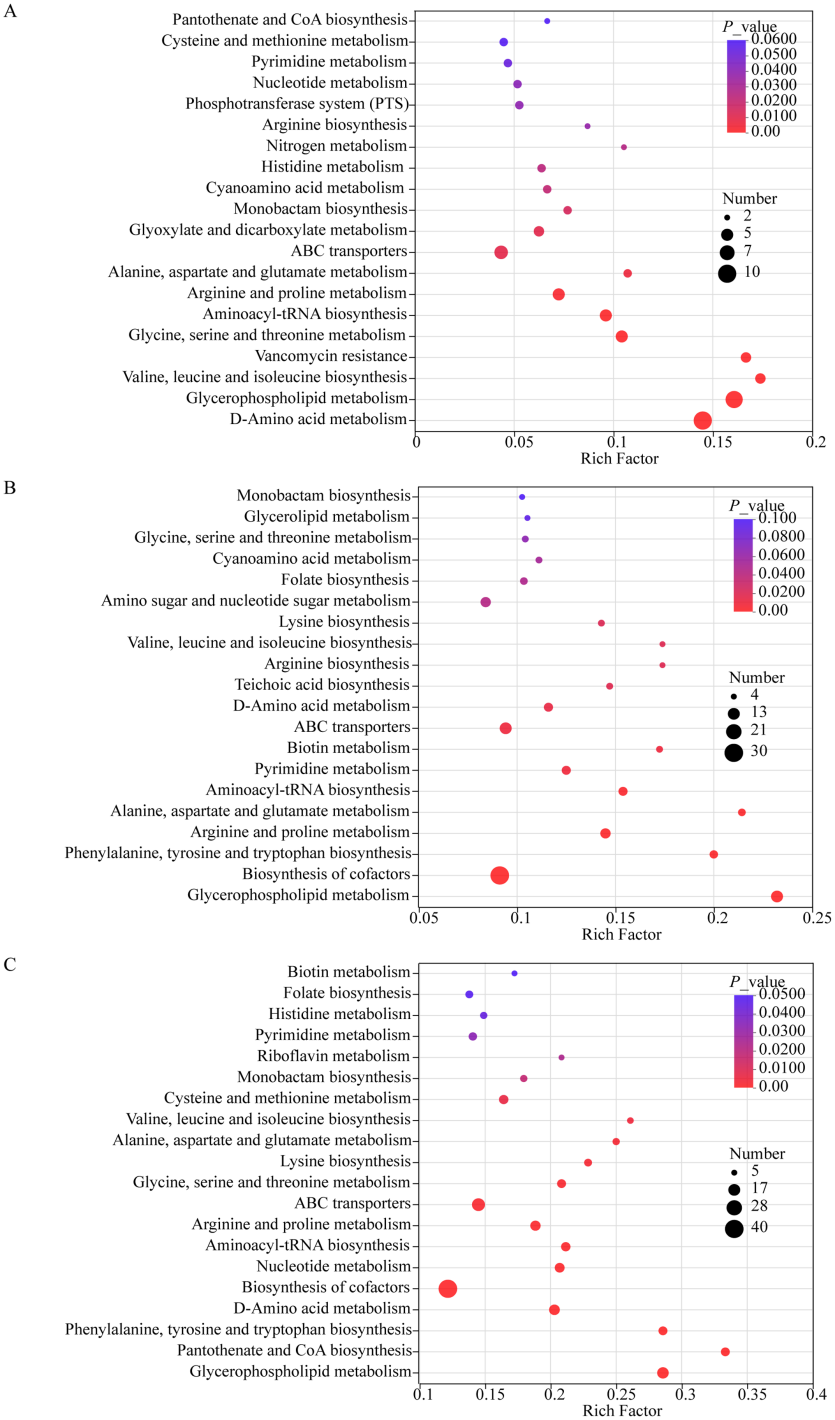
Metabolic pathway impact analysis of differentially expressed metabolites following 7 day refrigerated storage. **(A–C)** Enrichment bubble plots showing significantly altered metabolic pathways in **(A)** *B. lactis* 23426 co-culture, **(B)** *B. bifidum* 91 co-culture, and **(C)** *B. longum* BB68S co-culture compared to HYY. All analyses were performed using MetaboAnalyst 5.0 with the KEGG pathway database, considering metabolites with VIP > 1.0 and *p* < 0.05 as statistically significant. The *x-ax*is represents the pathway impact score calculated based on topological centrality, while the *y*-axis shows −log_10_ (*p*-value) from Fisher’s exact test. The bubble size represents pathway impact value, while color intensity indicates −log_10_ (*p*-value).

Elucidation of the metabolic mechanisms behind *Bifidobacterium*-mediated suppression of post-acidification over a 7-day refrigerated storage period was achieved through pathway topology analysis and betweenness centrality assessment. Comparative analysis revealed consistent downregulation of core metabolic pathways across all *Bifidobacterium*-supplemented yogurts versus HYY yogurt (Figure 7). Pathway suppression of significant magnitude (VIP ≥ 1, *p* < 0.05) was identified in several yogurt formulations. In 23426 + HYY yogurt, the pathways exhibiting notable suppression included D-amino acid metabolism, glycerophospholipid metabolism, valine/leucine/isoleucine biosynthesis, glycine/serine/threonine metabolism, aminoacyl-tRNA biosynthesis, and arginine/proline metabolism (Figure 7A). For the 91 + HYY yogurt, the affected pathways consisted of glycerophospholipid metabolism, pantothenate/CoA biosynthesis, phenylalanine/tyrosine/tryptophan biosynthesis, D-amino acid metabolism, and cofactor biosynthesis (Figure 7B). In the case of BB68S + HYY yogurt, significant pathway suppression was noted for glycerophospholipid metabolism, cofactor biosynthesis, phenylalanine/tyrosine/tryptophan biosynthesis, arginine/proline metabolism, alanine/aspartate/glutamate metabolism, and aminoacyl-tRNA biosynthesis (Figure 7C).

Systemic downregulation of glycerophospholipid metabolism indicates its critical role in post-acidification mitigation. As essential regulators of lactic acid bacterial (LAB) membrane fluidity and proton motive force, reduced glycerophospholipid biosynthesis compromises membrane integrity, limiting LAB metabolic activity and lactic acid accumulation. This aligns with established *bifidobacteria*-derived metabolite effects (e.g., acetate, formate) on pH-dependent lipid remodeling (Qiao et al., 2025; Lou et al., 2024). The inhibition of amino acid biosynthesis pathways, including branched-chain and aromatic amino acids, is associated with a decrease in proteolytic activity, which is a key driving factor of post-fermentation acidification (Shori, 2020). Notably, LAB rely on extracellular peptidases to hydrolyze casein into free amino acids, which are then further metabolized into ammonia to neutralize the acidic environment. The downregulation of these pathways in yogurt enriched with *bifidobacteria* suggests a metabolic shift toward carbon metabolism suppression, reallocating resources from nitrogen metabolism to carbohydrate utilization—a mechanism previously observed in synthetic biological systems (Yang et al., 2023). Moreover, the inhibition of cofactor biosynthesis (such as pantothenic acid and riboflavin) in both groups (Figures 7B,C) may impair the redox balance within LAB. Depletion of NADH/FADH₂ cofactors impairs lactate dehydrogenase activity, reducing glycolytic flux and acidogenesis (Yan et al., 2021). *In vitro* evidence indicates *bifidobacterial* exopolysaccharides chelate Mg^2+^/Mn^2+^ ions required for cofactor synthesis, indirectly inhibiting LAB metabolism, which supports this hypothesis (Xu et al., 2019; Xu et al., 2021; Kant Bhatia et al., 2021). The reduction in *B. longum* BB68S and *B. bifidum* 91 abundance primarily stems from the decreased expression of glycerophospholipid metabolism, cofactor biosynthesis, and amino acid metabolic pathways. Conversely, the variation in *B. animalis* 23426 abundance is associated with D-amino acid metabolism and valine/leucine/isoleucine biosynthesis.

## 4 Conclusion

This study demonstrates that *Bifidobacterium* supplementation significantly influences both the fermentation dynamics and storage stability of yogurt through distinct metabolic mechanisms. During co-fermentation, *Bifidobacterium* strains exhibited two primary effects: (1) they accelerated acid production after the initial 2-h lag phase, reducing time to fermentation endpoint (70 °T) by 0.5–2 h through enhanced galactose metabolism and provision of amino acids/peptides that stimulated *S. thermophilus* proliferation during logarithmic growth; and (2) they competitively inhibited *L. bulgaricus*, reducing its final viability by 0.2–0.8 lg CFU/mL. During refrigerated storage, *Bifidobacterium* modulated post-fermentation acidification through three key metabolic adaptations: (1) downregulation of glycerophospholipid metabolism, (2) reduced catabolism of branched-chain and aromatic amino acids, and (3) inhibition of pantothenic acid cofactor synthesis. These metabolic shifts resulted in a significantly lower acid accumulation (*Δ* 3.08–7.49 °T) compared to conventional yogurt (Δ 9.42 °T) over 21 days of storage. These findings highlight the dual-phase benefits of *Bifidobacterium* supplementation: improving fermentation efficiency while enhancing storage stability. However, the strain-specific nature of these effects necessitates careful selection of *Bifidobacterium* strains for optimal technological performance. The results provide a scientific basis for developing next-generation probiotic yogurts with controlled acidification profiles and improved shelf-life characteristics.

## Data Availability

The original contributions presented in the study are included in the article/Supplementary material, further inquiries can be directed to the corresponding authors.

## Footnotes

1
https://cloud.majorbio.com

2
https://www.metaboanalyst.ca
